# Supplementation of Medium-Chain Triglycerides Combined with Docosahexaenoic Acid Inhibits Amyloid Beta Protein Deposition by Improving Brain Glucose Metabolism in APP/PS1 Mice

**DOI:** 10.3390/nu15194244

**Published:** 2023-10-01

**Authors:** Zehao Wang, Dalong Zhang, Cheng Cheng, Zhenzhen Lin, Dezheng Zhou, Yue Sun, Wen Li, Jing Yan, Suhui Luo, Zhiyong Qian, Zhenshu Li, Guowei Huang

**Affiliations:** 1 Department of Nutrition and Food Science, School of Public Health, Tianjin Medical University, Tianjin 300070, China; 13103593666@163.com (Z.W.); chengcheng@tmu.edu.cn (C.C.); zzlin@tmu.edu.cn (Z.L.); dezhengzhou@163.com (D.Z.); sunyuejy163@163.com (Y.S.); liwen828@tmu.edu.cn (W.L.); luosuhui@tmu.edu.cn (S.L.); 2 Department of Toxicology, Tianjin Centers for Disease Control and Prevention, Tianjin 300011, China; zhangdalone@126.com (D.Z.); tjcdcdl@163.com (Z.Q.); 3 Tianjin Key Laboratory of Environment, Nutrition and Public Health, Tianjin 300070, China; yanjing@tmu.edu.cn; 4 Department of Social Medicine and Health Administration, School of Public Health, Tianjin Medical University, Tianjin 300070, China; 5 Department of Critical Care Medicine and Anesthesiology, Tianjin Medical University General Hospital, Tianjin 300052, China

**Keywords:** medium-chain triglycerides, docosahexaenoic acid, Alzheimer’s disease, amyloid beta protein, ^18^F-FDG-PET-CT, glucose metabolism, APP/PS1 mouse

## Abstract

The deterioration of brain glucose metabolism predates the clinical onset of Alzheimer’s disease (AD). Medium-chain triglycerides (MCTs) and docosahexaenoic acid (DHA) positively improve brain glucose metabolism and decrease the expression of AD-related proteins. However, the effects of the combined intervention are unclear. The present study explored the effects of the supplementation of MCTs combined with DHA in improving brain glucose metabolism and decreasing AD-related protein expression levels in APP/PS1 mice. The mice were assigned into four dietary treatment groups: the control group, MCTs group, DHA group, and MCTs + DHA group. The corresponding diet of the respective groups was fed to mice from the age of 3 to 11 months. The results showed that the supplementation of MCTs combined with DHA could increase serum octanoic acid (C8:0), decanoic acid (C10:0), DHA, and β-hydroxybutyrate (β-HB) levels; improve glucose metabolism; and reduce nerve cell apoptosis in the brain. Moreover, it also aided with decreasing the expression levels of amyloid beta protein (Aβ), amyloid precursor protein (APP), β-site APP cleaving enzyme-1 (BACE1), and presenilin-1 (PS1) in the brain. Furthermore, the supplementation of MCTs + DHA was significantly more beneficial than that of MCTs or DHA alone. In conclusion, the supplementation of MCTs combined with DHA could improve energy metabolism in the brain of APP/PS1 mice, thus decreasing nerve cell apoptosis and inhibiting the expression of Aβ.

## 1. Introduction

Alzheimer’s disease (AD) is a chronic neurodegenerative disease characterized by progressive cognitive impairment, and it is considered as one of the primary etiologies of dementia in the elderly population [1]. Amyloid beta protein (Aβ) is one of the major pathological features of AD [2]. According to the amyloid cascade hypothesis, Aβ is generated from the amyloid precursor protein (APP) after, at first, cleavage at the β, and then at the γ cleavage sites. The β-site APP cleaving enzyme-1 (BACE1) is viewed as the major β-secretase, while presenilin-1 (PS1) is the main component of γ-secretase [3]. The accumulation of Aβ occurs within nerve cells and subsequently forms plaques outside the cells, which leads to synaptic dysfunction, cell apoptosis, and progressive cognitive decline [4]. The clearance of Aβ from the brain becomes a main therapeutic strategy for AD [5].

Drugs approved for treating AD primarily target symptomatic relief without providing a cure or preventive measures against the underlying disease [6]. Thus, treatments that have potential to delay or prevent the progression of AD are urgently needed [7]. Epidemiological evidence has suggested that low-risk and cost-effective dietary interventions are excellent strategies through which to mitigate the impairments associated with aging and neurodegenerative diseases [8,9]. Currently, medium-chain triglycerides (MCTs) and docosahexaenoic acid (DHA) have shown remarkable effectiveness and have garnered significant attention [10,11].

MCTs, which are composed of medium-chain fatty acids (MCFAs) and glycerol [12], typically contain a mixture of 6–12 carbon saturated fatty acids, with octanoic acid (C8:0) and decanoic acid (C10:0) being the dominant species [13]. MCTs are ketogenic substances, of which β-hydroxybutyrate (β-HB) makes up about 70% of the circulating ketone pool [14]. β-HB can cross the blood–brain barrier and can be consumed in the brain when glucose is limited [15]. Previous studies have suggested that the elevation of brain ketone levels through the intake of MCTs may reduce cerebral Aβ deposition and improve cognitive function [16]. For instance, MCTs exhibit beneficial effects on cognitive abilities in AD patients with APOE4^−/−^, and the therapeutic impact of MCTs may be attributed to their ketogenic effects [10]. Meanwhile, it has been confirmed that the peripheral administration of ketones could significantly reduce Aβ expression levels, as well as improve learning and memory ability in APP mice by blocking intracellular Aβ_42_ accumulation [17].

DHA, as an essential omega-3 fatty acid, is a structural component of the membranes in the central nervous system [18]. Previous studies have shown a variety of mechanisms through which DHA may be important for brain structure and function, including maintaining the flexibility/compressibility of membrane lipids, improving mitochondrial dysfunction and neuroinflammation, and in increasing brain energy uptake [19]. Meanwhile, adequate animal studies and clinical trials have shown that the dietary supplementation of DHA could significantly increase the levels of DHA in the brain [20,21,22]. Recent evidence has indicated that the consumption of DHA derived from fish is associated with enhanced cognitive function and a decreased risk of AD [23]. Pifferi et al. demonstrated that, in non-human primate Microcebus murinus, DHA increased brain glucose uptake and metabolism, as well as concomitantly reduced anxiety [24]. In a randomized controlled trial study, daily oral DHA supplementation (2 g/day) for 24 months improved cognitive function and changed blood biomarker-related Aβ-mediated autophagy in people with mild cognitive impairment [25]. These promising results suggested that DHA supplementation may be a viable solution through which to mitigate the detrimental effects of Aβ plaque accumulation in the brain.

As the causes and processes of AD are continuously explored, brain energy deficiency and metabolic alterations have attracted attention [26]. Previous studies found that, as the bioenergetic basis for neurotransmission, the deterioration of brain glucose metabolism is progressive and regional, and that it precedes symptoms and accelerates the apoptosis of nerve cells and the deposition of Aβ [27]. Therefore, the strategies of “brain energy rescue” have garnered significant attention [28]. These strategies are aimed at increasing the brain energy level, favoring cell energy restoration, and decreasing the levels of Aβ expression [29]. For MCTs, the brain’s ability to use ketones is significant when glucose availability is limited [30], and the uptake of ketones by the brain appears to remain unaffected, unlike glucose, which is often impaired in AD patients [31]. For DHA, the pathways through which to improve brain glucose metabolism include affecting blood–brain barrier glucose transport, neuronal glucose transport, and brain energy metabolism [24]. In summary, both MCTs and DHA exhibit functions in improving brain energy metabolism and inhibiting Aβ accumulation.

The present study was designed to investigate the effects of the supplementation of MCTs combined with DHA on nerve cell apoptosis and the expression levels of Aβ, APP, BACE1, and PS1 by improving brain glucose metabolism in the APPswe/PS1De9 (APP/PS1) AD model mice. The present study hypothesized that the supplementation of MCTs combined with DHA significantly decreases nerve cell apoptosis and the expression levels of AD-related proteins by improving brain glucose metabolism.

## 2. Materials and Methods

### 2.1. Animals and Dietary Treatment

All procedures were approved by the Tianjin Medical University Animal Ethics Committee (TMUaMEC2022046). Male APP/PS1 mice with a C57BL/6J background were purchased from Cavins Laboratories (Changzhou, China). After acclimatization for two weeks, 3-month-old APP/PS1 mice were randomly divided into four different diet groups (15 mice/group): (1) the MCTs group (MCTs) was fed with the 100 g MCTs/kg supplemented diet (equivalent 42.5 g/d MCTs for humans); (2) the DHA group (DHA) was fed with the 2.91 g DHA/kg supplemented diet (80.8% purity, equivalent 1.00 g/d DHA for humans); (3) the MCTs + DHA group (MCTs + DHA) was fed with the 2.91 g DHA/kg and 100 g MCTs/kg supplemented diet (equivalent 42.5 g/d MCTs and 1.00 g/d DHA for humans); (4) the Control diet group (Control) was fed with the regular diet. The control diet was modified from AIN-93M. Soybean oil was replaced with corn oil in the AIN-93M to minimize the effect of α-linolenic acid (ALA) in the experiment. The supplements were added to the diet by mixing 100 g/kg MCT oil and/or 2.91 g/kg DHA oil during the feed-making process. The MCT oil added to the diet was obtained from Nisshin OilliO Co., Ltd. (Shanghai, China) and comprised mainly of 73.7% C8:0 and 26.0% C10:0. The DHA oil added to the diet was obtained from Acmec Biochemical Co., Ltd. (Shanghai, China), and the form was non-esterified DHA (80.8% purity). The control diet had the same components as the diet of the supplementation group, except that no additional intervention was added. The energy balance of the diet mainly depends on the adjustment of corn oil content. Refer to Table S1 for detailed information on the macro and micronutrient composition of AIN-93M and Table S2 for the fatty acid profile of the corn oil. All of the mice in the study were individually housed in a specific pathogen-free (SPF) facility under a controlled temperature (24 ± 2 °C) environment with a 12 h light/dark cycle that allowed for free access to food and water until sacrifice. The dietary treatment was initiated at the 3-month-old stage, and the supplementation lasted for 8 months. The mice were anesthetized by carbon dioxide inhalation and sacrificed by cervical dislocation at the 11-month-old stage, and the brain tissue (the cerebellum was removed) was either fixed with 4% paraformaldehyde (PFA) for immunohistochemical staining or stored at −80 °C after liquid nitrogen flash-freezing. The mice blood was centrifuged immediately, and the serum obtained was stored at −80 °C for subsequent assays.

### 2.2. Gas Chromatograph-Mass Spectrometer (GC-MS)

The levels of fatty acids in the serum of mice after supplementation were measured by GC-MS. During the pre-treatment phase, 500 μL of standard fatty acid methyl ester was taken and mixed with 25 μL of 500 ppm methyl n-19 acid as the internal standard. The mixed standard product was added into the sample bottle and detected by Agilent 7890/5975C GC-MS. Before the analysis, 100 μL of mice serum was thawed on ice and mixed with 1 mL of a chloroform–methanol solution; this was then ultrasonicated for 30 min to collect the supernatant. Then, the sample was mixed with 2 mL of a 1% sulfuric acid–methanol solution and heated in an 80 °C -water bath for 30 min to produce methyl esters. Finally, 1 mL of an n-hexane extraction was added and washed with 5 mL of pure water. A 500 mL volume of the supernatant was then combined with 25 mL of methyl n-19thenate as an internal standard, and this was then mixed before being added to a sample bottle for GC-MS analysis. For gas chromatography separation, the injection volume was set at 1 μL with a split ratio of 10:1. The samples were separated by Agilent DB-WAX capillary column (30 m × 0.25 mm, ID × 0.25 μm) gas chromatography. Helium was used as the carrier gas with a flow rate of 1.0 mL/min. The mass spectra were obtained using Agilent 7890/5975C gas-mass spectrometer with an ionization source temperature of 230 °C and a transmission line temperature of 250 °C. The electron bombardment ionization source was used with the SIM scanning mode, with an electron energy of 70 eV. MSD Chem Station software (E.02.00 SP2 05/09) was used to extract the chromatographic peak area, and the retention time determination was conducted to calculate the content of chain fatty acids in the samples.

### 2.3. Immunohistochemical Staining

The AD-related protein (Aβ, APP, BACE1, and PS1) expressions were quantified by immunohistochemical staining. The brain tissue was fixed in 4% PFA, processed for paraffin embedding, and sliced into 5 μm thickness sections. During immunohistochemistry experiments, tissue slices were dewaxed, rehydrated, rinsed, and then repaired by citric acid antigens, and this was performed following the inactivation of endogenous peroxidase with 3% H_2_O_2_ for 15 min and cellular permeabilization with 0.5% Triton-X for 30 min. Then, an ABC-blocking serum working solution (PK-6105, Vector Lab, Newark, CA, USA) was used to block non-specific antibody binding sites. Based on the specific experimental objectives, different primary antibodies were chosen and incubated overnight at 4 °C: Aβ (1:1000, ab201060, Abcam, Waltham, MA, USA), APP (1:1000, ab32136, Abcam), BACE1 (1:1000, bs-0164R, Bioss, Beijing, China), and PS1 (1:1000, ab76083, Abcam). Biotin-conjugated secondary antibodies were used for binding at 37 °C for 30 min, and the ovalbumin–biotin–enzyme complex was used to combine with a 3,3′-Diaminobenzidine (DAB) substrate (zli-9018, ORIGENE, Rockville, MD, USA) at 23 °C for 5 min. Hematoxylin (EE-0012, Spark Jade, Beijing, China) was used for nuclear staining, and tap water was used to maintain the blue color. Finally, a gradient of high-percentage ethanol was used for dehydration, xylene was used for transparency, and neutral resin adhesive was used to seal the slices. The images were obtained with a microscope (Olympus, Tokyo, Japan), and the integrated optical density (IOD) of each was determined with Image-Pro Plus Version 6.0 image analysis software.

### 2.4. TdT-Mediated dUTP Nick End Labeling (TUNEL Assay)

When genomic DNA is broken, the exposed 3′-OH ends can bind to fluorescein-labeled dUTP in the presence of terminal deoxynucleotidyl transferase (TdT), which can be detected using fluorescence microscopy. Tissue slices were deparaffinized with xylene, hydrated with a gradient of high percentage ethanol to distilled water, blocked with 20 μg/mL of a proteinase K solution for 30 min, processed with 0.5% Triton-X for 30 min, and then incubated with the rTDT reaction system (G3250, Promega, Shanghai, China) for 60 min at 37 °C in the dark. The reaction was then terminated using a 2× sodium citrate buffer for 15 min to terminate the reaction. Finally, 4′,6-diamidino-2phenylindole (DAPI) staining (H-1800, Vector Laboratories, USA) was used to define the nuclear area. The ratio of the number of apoptotic cells (green) and DAPI fluorescent spots (blue) was calculated by the Olympus IX81 microscope (Olympus, Japan).

### 2.5. Enzyme-Linked Immunosorbent Assay (ELISA)

ELISA is a widely used immunoenzymatic technique for quantifying the concentration of a specific antigen. In the present study, a β-Hydroxybutyrate Colorimetric Assay Kit (E-BC-K785-M, Elabscience, Wuhan, China) was used to measure the levels of β-HB in the mice serum and brain samples. For the mice serum samples, the upper layer of fluid was aspirated by centrifugation at 4 °C; as for the brain tissue samples, 30 mg of brain tissue was taken and centrifuged with phosphate buffer saline (PBS) to extract the upper fluid, and the protein levels were quantified with a BCA protein assay kit in accordance with the manufacturer’s instructions. The standard solution was added into the blank hole of the planned enzyme label plate according to the gradient concentration and then incubated for 30 min at 37 °C. Then, the plate was washed and an enzyme-labeled reagent was added for 30 min at 37 °C. Finally, color-developing agents A and B were added to each hole and reacted for 10 min at 37 °C in the dark. The plotted values were measured and analyzed by an enzyme marker (Bio-Tek SynergyMx, Santa Clara, CA, USA).

### 2.6. ^18^F-Fluoro-2-deoxyglucose Integrated PET-CT System Imaging

It is known that ^18^F-fluoro-2-deoxyglucose (^18^F-FDG) positron emission tomography (PET) is commonly used to measure the cerebral metabolic rate of glucose (CMRglc). In the present study, a PET-CT instrument (Inliview-3000B, Novel Medical, Beijing, China) was used to explore the improvement of brain glucose metabolism in mice after dietary intervention. After injection, ^18^F-FDG was rapidly distributed throughout the mice’s bodies, and positron imaging was then, usually, performed for 35 to 40 min. Before the injection, the radioactive activity was maintained within 130–170 μCi by diluting the initial 200 μCi. Mice were weighed on-site and anesthetized with atomized 0.5% isoflurane before injection and during the experiment. The mice image and analysis were performed by PMOD v3.9 (PMOD Technologies, Zürich, Switzerland). The construction parameters of the CT images were as follows: 512 × 512 × 500 matrices with a 0.13672 mm pixel size. The construction parameters of the PET image are as follows: 140 × 140 × 112 matrices (with a 0.500 mm pixel size). The software can directly export PET-CT combination maps. The formula used for standardized uptake value (SUV) and glucose-corrected standardized uptake value (SUVglc) were as follows: SUV = average tissue active concentration (kBq/cc) × body weight (g)/injection dose (kBq); SUVglc = SUV × blood glucose level (mg/dL).

### 2.7. Statistical Analysis

The data were expressed as the mean ± standard deviation (SD). Comparisons among the different groups were performed by one-way ANOVA. A pairwise comparison between the means of the different groups was performed with the SNK-q test. The statistical software package SPSS 25.0 (IBM Corp., Armonk, NY, USA) was used to evaluate differences among groups, and a *p* value less than 0.05 was considered statistically significant. GraphPad Prism 9.0 (GraphPad Software Inc., San Diego, CA, USA) was used to draw the statistical graphics.

## 3. Results

### 3.1. Supplementation of the MCTs Combined with DHA Increased Serum C8:0, C10:0, and DHA Levels in APP/PS1 Mice

**Figure 1:**
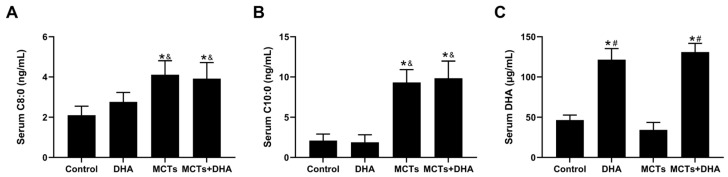
Supplementation of medium-chain triglycerides (MCTs) combined with docosahexaenoic acid (DHA) increased the serum C8:0, C10:0, and DHA levels in APP/PS1 mice. The 10-week-old APP/PS1 mice were assigned into four groups in equal numbers (15 mice/group): (1) the DHA group (DHA) were fed with a 2.91 g DHA/kg supplemented diet; (2) the MCTs group (MCTs) were fed with a 100 g MCTs/kg supplemented diet; (3) the MCTs + DHA group (MCTs + DHA) were fed with a 2.91 g DHA/kg and 100 g MCTs/kg supplemented diet; and (4) the Control group (Control) were fed with a regular diet. The dietary intervention began at 3 months of age and ended at 11 months, with intervention running in total for 8 months. (**A**) The level of octanoic acid (C8:0) in the serum of mice. (**B**) The level of decanoic acid (C10:0) in the serum of mice. (**C**) The level of DHA in the serum of mice. The plotted values were expressed as the mean ± SD (*n* = 6 mice/group). *: *p* < 0.05 compared with Control group. &: *p* < 0.05 compared with DHA group. #: *p* < 0.05 compared with MCTs group.

The main components of the MCTs (C8:0 and C10:0) and DHA were detected after dietary intervention by GC-MS. The serum C8:0 and C10:0 levels were higher in the MCTs group and the MCTs + DHA group compared with the Control group and the DHA group (*p* < 0.05, Figure 1A,B). However, there were no significant differences in the C8:0 and C10:0 levels between the Control group and DHA group, nor between the MCTs group and MCTs + DHA group. The serum DHA levels were higher in the DHA group and the MCTs + DHA group compared with the Control group and MCTs group (*p* < 0.05, Figure 1C). However, there were no significant differences in the DHA levels between the Control group and MCTs group, nor between the DHA group and the MCTs + DHA group. These results showed that MCT and DHA supplementation increased the serum C8:0, C10:0, and DHA levels, respectively. Moreover, the combined supplementation of MCTs with DHA also increased the serum C8:0, C10:0, and DHA levels.

### 3.2. Supplementation of MCTs Combined with DHA Decreased AD-Related Proteins Expression Levels in Brain of APP/PS1 Mice

**Figure 2:**
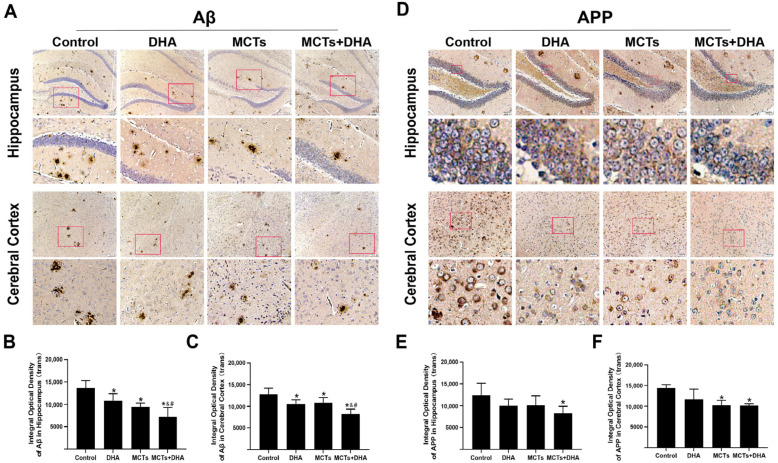
Supplementation of MCTs combined with DHA decreased the amyloid beta protein (Aβ) and amyloid precursor protein (APP) expression levels in the cerebral cortex and hippocampus of APP/PS1 mice. Mice were fed as described in Figure 1. Representative micrographs of the 3,3′-Diaminobenzidine (DAB) staining of Aβ in the hippocampus and cerebral cortex. Brown diffuse plaques staining indicated the presence of Aβ. Scale bar = 100 μm (**A**). Quantitative analysis of the levels of Aβ expression in the hippocampus (**B**) and cerebral cortex (**C**). Representative micrographs of the DAB staining of APP in the hippocampus and cerebral cortex. APP presented as brown granules in staining. Scale bar = 50 μm (**D**). Quantitative analysis of the levels of APP expression in the hippocampus (**E**) and cerebral cortex (**F**). Each below merge column depicted a magnified image of the rectangular region of its corresponding image in the upper merge column. The plotted values were expressed as the mean ± SD (*n* = 5 mice/group). *: *p* < 0.05 compared with the Control group. &: *p* < 0.05 compared with the DHA group. #: *p* < 0.05 compared with the MCTs group.

The levels of the AD-related protein expressions in the cerebral cortex and hippocampus were detected by immunohistochemical analysis. Immunohistochemical analysis of the Aβ expression showed that the Aβ expression levels in the hippocampus and cerebral cortex of the DHA group, MCTs group, and MCTs + DHA group were lower than those in the Control group (*p* < 0.05, Figure 2A–C). The levels in the MCTs + DHA group in the hippocampus and cerebral cortex were lower than those in the other dietary intervention groups (*p* < 0.05, Figure 2A–C). There were no significant differences between the DHA group and MCTs group in the hippocampus and cerebral cortex (*p* > 0.05, Figure 2A–C).

APP has been extensively studied for its role as the precursor of Aβ in Alzheimer’s disease. To test whether a supplementation of MCTs combined with DHA could decrease the expression of APP, the levels of APP were assessed. As shown in Figure 2, the APP expression level in the hippocampus of the MCTs + DHA group was lower than the Control group (*p* < 0.05, Figure 2D,E). Although the levels of APP in the hippocampus of the DHA group and MCTs group were lower than the Control group, there were no significant differences between them (*p* > 0.05, Figure 2D,E). Meanwhile, the levels of APP in the cerebral cortex of the MCTs group and MCTs + DHA group were lower than the Control group (*p* < 0.05, Figure 2D,F). However, the DHA group only showed a reduction without significant differences in the cerebral cortex (*p* > 0.05, Figure 2D,F).

**Figure 3:**
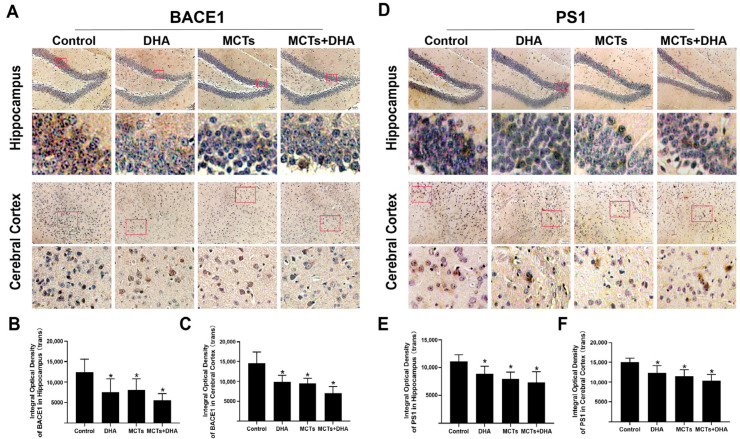
Supplementation of MCTs combined with DHA decreased the β-site APP cleaving enzyme-1 (BACE1) and presenilin-1 (PS1) expression levels in the cerebral cortex and hippocampus of the APP/PS1 mice. Mice were fed as described in Figure 1. Representative micrographs of the DAB staining of BACE1 in the hippocampus and cerebral cortex. Brown granules represented BACE1 in staining. Scale bar = 50 μm (**A**). Quantitative analysis of the levels of BACE1 expression in the hippocampus (**B**) and cerebral cortex (**C**). Representative micrographs of DAB staining of PS1 in the hippocampus and cerebral cortex. Brown granules represented PS1 in staining. Scale bar = 50 μm (**D**). Quantitative analysis of the levels of PS1 expression in the hippocampus (**E**) and cerebral cortex (**F**). Each below merge column depicted a magnified image of the rectangular region of its corresponding image in the upper merge column. The plotted values were expressed as the mean ± SD (*n* = 5 mice/group). *: *p* < 0.05 compared with the Control group.

To investigate whether the cleavage of APP is associated with the BACE1 expression and whether the BACE1 expression was modulated by MCTs and DHA, we assessed the immunohistochemical staining for BACE1 in the hippocampus and cerebral cortex of the APP/PS1 mice. As the results show in Figure 3, the BACE1 expression level of the DHA group, MCTs group, and the MCTs + DHA group were lower than the Control group in the hippocampus and cerebral cortex (*p* < 0.05, Figure 3A–C). In addition, the levels of BACE1 expression in the hippocampus and cerebral cortex of the MCTs + DHA group were lower but did not differ significantly from the MCTs and DHA alone (*p* > 0.05, Figure 3A–C).

Generation of Aβ requires a second cleavage by γ-secretase. As the main component of γ-secretase, the level of PS1 expression was detected. As shown in Figure 3, PS1 expression levels in the hippocampus and cerebral cortex of the DHA group, MCTs group, and the MCTs + DHA group were lower than those in the Control group (*p* < 0.05, Figure 3D–F). Moreover, there were no significant differences in the hippocampus and cerebral cortex between the intervention groups (*p* > 0.05, Figure 3D–F). Taken together, the above results showed that the supplementation of MCTs and DHA inhibited the Aβ deposition and decreased the levels of APP, BACE1, and PS1 expression in the hippocampus and cerebral cortex of the APP/PS1 mice. Furthermore, the supplementation of MCTs combined with DHA was more effective than DHA or MCTs alone.

### 3.3. Supplementation of MCTs Combined with DHA Decreased the Apoptosis of Nerve Cells in the Cerebral Cortex and Hippocampus of APP/PS1 Mice

**Figure 4:**
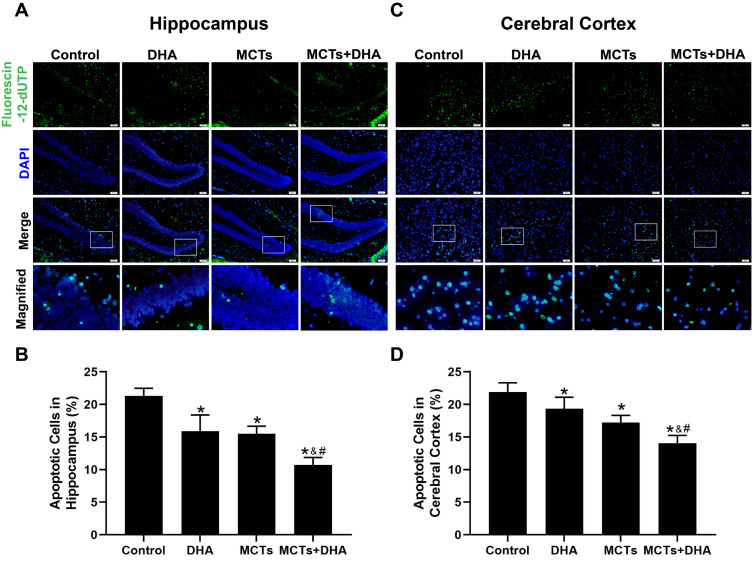
Supplementation of MCTs combined with DHA decreased the nerve cell apoptosis of APP/PS1 mice. Mice were fed as described in Figure 1. Representative micrographs of nerve cell apoptosis in the hippocampus, in which apoptotic cells are stained with Fluorescein-12-dUTP (green) and DAPI (blue). Scale bar = 50 μm (**A**). Quantitative analysis of nerve cell apoptosis in the hippocampus (**B**). Representative micrographs of nerve cell apoptosis in the cerebral cortex, in which apoptotic cells are stained with Fluorescein-12-dUTP (green) and DAPI (blue). Scale bar = 50 μm (**C**). Quantitative analysis of nerve cell apoptosis in the cerebral cortex (**D**). Each below merge column depicted a magnified image of the rectangular region of its corresponding image in the upper merge column. The plotted values were expressed as the mean ± SD (*n* = 5 mice/group). *: *p* < 0.05 compared with the Control group. &: *p* < 0.05 compared with the DHA group. #: *p* < 0.05 compared with the MCTs group.

To investigate whether the supplementation of MCTs combined with DHA could decrease the apoptosis of nerve cells, the TUNEL assay was used to assess the apoptosis rate in the hippocampus and cerebral cortex. The TUNEL assay revealed that the number of apoptotic cells in the Control group was the most among the four groups, and only a few apoptotic cells were observed in the DHA group, MCTs group, and MCTs + DHA group in the cerebral cortex and hippocampus (*p* < 0.05, Figure 4A–D), with the lowest apoptotic rate being found in the MCTs + DHA group. Additionally, the apoptosis rates were significantly lower in the MCTs + DHA group compared with the MCTs group and DHA group (*p* < 0.05, Figure 4A–D). These results showed that the supplementation of MCTs combined with DHA decreased the apoptosis of nerve cells. Moreover, the combined effect was significantly more beneficial than with MCTs or DHA alone, and the DHA group showed no significant differences from the MCTs group.

### 3.4. Supplementation of MCTs Combined with DHA Increased the Levels of β-HB in the Serum and Brain Tissue of APP/PS1 Mice

**Figure 5:**
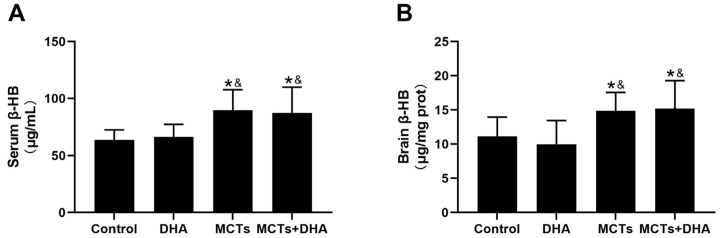
Supplementation of MCTs increased the β-hydroxybutyrate (β-HB) levels in the serum and brain tissue of APP/PS1 mice. Mice were fed as described in Figure 1. (**A**) Serum β-HB of the mice. (**B**) Brain tissue β-HB of the mice. The plotted values are expressed as the mean ± SD (*n*= 7 mice/group). *: *p* < 0.05 compared with the Control group. &: *p* < 0.05 compared with the DHA group.

The levels of β-HB in the MCTs + DHA group and MCTs group were higher than the DHA group and Control group, both in terms of in the serum and brain tissue (*p* < 0.05, Figure 5A,B). There were no significant differences between the Control group and DHA group, and the same was the case for the MCTs group and MCTs + DHA group. These results showed that the supplementation of MCTs or MCTs combined with DHA significantly increased the β-HB levels in the serum and brain tissue.

### 3.5. Supplementation of MCTs Combined with DHA Improved Glucose Metabolism in the Brain of the APP/PS1 Mice

**Figure 6:**
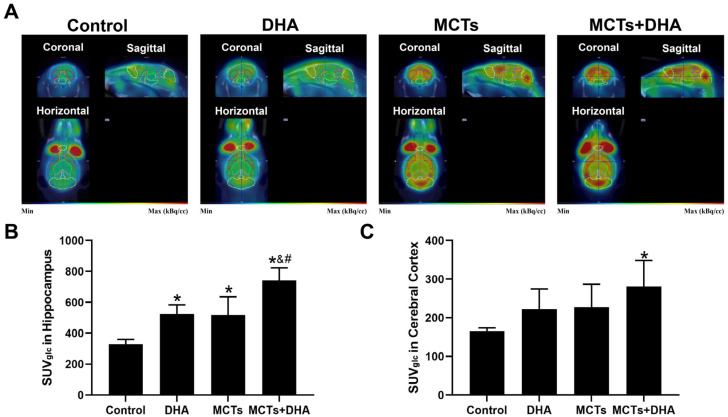
Supplementation of MCTs combined with DHA improved the glucose metabolism of APP/PS1 mice. Mice were fed as described in Figure 1. Representative ^18^F-fluoro-2-deoxyglucose integrated PET-CT (^18^F-FDG-PET-CT) isotope distribution map images in coronal, horizontal, and sagittal views (**A**). In the images, low isotope distribution regions displayed in the blue-green range and higher isotope distribution regions in the orange-red spectrum. Quantitative analysis of glucose-corrected standardized uptake value (SUVglc) in the hippocampus (**B**), and cerebral cortex (**C**). The plotted values are expressed as the mean ± SD (*n* = 5 mice/group). *: *p* < 0.05 compared with the Control group. &: *p* < 0.05 compared with the DHA group. #: *p* < 0.05 compared with the MCTs group.

The SUVglc was quantified to determine if a supplementation of MCTs combined with DHA could improve brain glucose metabolism with ^18^F-FDG-PET-CT. Representative images of coronal, sagittal, and horizontal views are shown in Figure 6A. The results showed that the SUVglc of the Control group was the lowest in the hippocampus and cerebral cortex among the four groups. Meanwhile, the SUVglc of MCTs + DHA was the highest, and the proportion was 2.24 and 1.70 times that of the Control group in the hippocampus and cerebral cortex, respectively. The SUVglc of the DHA group and MCTs group were higher in the hippocampus than in the Control group (*p* < 0.05, Figure 6A,B), and the SUVglc of the MCTs + DHA group was higher than in the Control group, DHA group, and MCTs group (*p* < 0.05, Figure 6A,B). For the cerebral cortex, only the MCTs + DHA group showed a significant improvement in the brain glucose metabolism (*p* < 0.05, Figure 6A,C). Moreover, there were no significant differences between the DHA group and MCTs group. The results showed that a supplementation of MCTs combined with DHA improved brain glucose metabolism. Additionally, a supplementation of MCTs combined with DHA was significantly more beneficial than MCTs or DHA alone.

## 4. Discussion

The present study showed that a supplementation of 100 g/kg MCTs combined with 2.91 g/kg DHA for 8 months significantly improved the brain glucose metabolism, as well as decreased the nerve cell apoptosis and AD-related protein (Aβ, APP, BACE1, and PS1) expression levels in the cerebral cortex and hippocampus of the 11-month-old APP/PS1 mice. Meanwhile, the results showed that a supplementation of MCTs combined with DHA was more efficient than MCTs or DHA alone.

Aβ, a major component of senile plaques (and which are one of the pathological hallmarks of AD), plays a critical role in the progression of Alzheimer’s disease [32]. According to the Aβ production pathway, its overproduction and deposition are closely related to the levels of certain proteins (APP, PS1, and BACE1) [33]. APP, as the Aβ precursor protein, is processed by two proteolytic pathways: non-amyloid-producing (mainly α-secretase) and amyloid-producing (mainly β-secretase). Usually, the non-amyloidosis pathway is dominant [34]. However, increased BACE1 expression promotes APP to be processed by the β-secretase pathway rather than the α-secretase pathway, thus resulting in more Aβ production [35]. In the amyloidosis pathway, the β-secretase slits APP at the N-terminal of Aβ (Asp1β site), thereby releasing the N-terminal fragment of APP and 99 amino acids APP-CTF (C99 or CTFβ). Subsequently, γ-secretase (mainly PS1) cleaves C99 in the transmembrane domain to release Aβ [36]. Mao et al. showed that mitochondrial targeting catalase could reduce Aβ production in APP mice by reducing abnormal APP and BACE1 levels [37]. In the present study, a supplementation of MCTs combined with DHA decreased the levels of related protein expression, such as APP, BACE1, and PS1; furthermore, it was also found that it restricted the β-secretase cleavage pathway and inhibited the production of Aβ.

In previous studies, MCTs and DHA have extensively been used to decrease the expression of Aβ and nerve cell apoptosis. Mett et al. demonstrated that the protective effect of MCFA might not only be based on improving energy metabolism, but also on a stimulation of Aβ degradation in the brain [38]. In addition, some studies have suggested that β-HB treatment could prevent the overload of autophagosomes and improve neuronal survival under energy-deficient conditions, as the ATP levels of the cells are better preserved [39]. In the present study, a supplementation of MCTs decreased the level of Aβ expression and nerve cell apoptosis, which is consistent with the above findings. As for the benefits of DHA, Green et al. demonstrated that a dietary supplementation of DHA reduced the intraneuronal accumulation of Aβ and tau by reducing the homeostasis level of PS1 in the 3×Tg-AD mice [40]. Meanwhile, additional research has revealed that DHA could decrease neuronal cell apoptosis [41]. In the present study, DHA supplementation decreased the level of Aβ expression and nerve cell apoptosis, which was consistent with the above findings. Furthermore, the supplementation of MCTs combined with DHA achieved superior outcomes compared with MCTs or DHA alone.

Impaired brain glucose metabolism appears to be a central hallmark of neurodegenerative diseases of aging, and it precedes the onset of clinical symptoms in AD [26]. The previous PET studies demonstrated that certain cortex regions have a 10–12% deficit in glucose uptake in mild cognitive impairment, and this defect becomes more widespread with the onset of AD and worsens during its progression [28]. When brain glucose metabolism decreases, neuronal death and neurotoxic protein accumulation accelerate, and the two reinforce each other, forming a vicious cycle [42]. In conclusion, the impairment of brain energy metabolism accelerated the neurotoxic protein accumulation and nerve cell apoptosis. The present study hypothesized that a supplementation of MCTs combined with DHA might inhibit the expression of Aβ by decreasing nerve apoptosis through improving glucose metabolism. Therefore, the brain glucose metabolism after dietary intervention was assessed.

^18^F-FDG-PET-CT, which is more effective than the standard PET, was used to evaluate the brain glucose metabolism of mice in the present study [43]. ^18^F-FDG is a glucose analog that structurally differs from glucose in that the radionuclide ^18^F replaces the hydroxyl group on the second carbon site. As a commonly used tracer in PET, it can be absorbed by neurons and captured due to the phosphorylation of hexokinase [44]. Since cells with more significant metabolic activity can take up and trap more ^18^F-FDG, the changes in neuronal and synaptic activity detected by ^18^F-FDG-PET provide a more accurate assessment of brain metabolism compared with the structural changes observed by CT or MRI scans [45]. These metabolic changes precede the structural changes in dementia [46]. In addition, ^18^F-FDG accumulates around synapses and represents local neuronal activity; moreover, it can be quantitatively analyzed for the most objective effect, providing superior diagnostic accuracy [43]. For example, a cohort of middle-aged individuals with dietary information and ^18^F-FDG-PCT scans were examined; following this, it was demonstrated that nutrients may play a role in glucose metabolism [47]. Another animal study indicated that a ketogenic diet increases brain glucose uptake in aged rats using ^18^F-FDG-PCT [48]. According to PET-CT experiments, the present study obtained the isotope intensity values of the hippocampus and cerebral cortex to observe the brain glucose metabolism. The results of the PET-CT experiments revealed that both MCTs and DHA exerted a protective effect on the brain glucose metabolism in different brain regions, including the hippocampus and cerebral cortex. Furthermore, the combined effect of MCTs with DHA was found to be significantly more beneficial than MCTs or DHA alone.

The strength of the present study is that PET-CT imaging was used to investigate the effects of MCTs and DHA on glucose metabolism. Meanwhile, the supplementation of MCTs combined with DHA was designed to explore whether its effect on reducing Aβ deposition was superior to that of single intervention. This study provided the theoretical basis for delaying the onset of AD or slowing its progression. However, this study has a limitation. The present study did not detect the brain levels of C8:0, C10:0, and DHA as these would consume large amounts of brain tissue, but the levels in the serum were detected as surrogate markers, thereby indicating the alterations of fatty acid levels in the mice.

## 5. Conclusions

The present study demonstrated that a supplementation of MCTs combined with DHA inhibited the Aβ deposition in 11-month-old APP/PS1 mice. The potential mechanism was that the combined intervention of MCTs and DHA improved brain glucose metabolism, thereby decreasing nerve cell apoptosis and the levels of APP, BACE1, and PS1 expression. These findings suggest that a supplementation of MCTs combined with DHA might be a therapeutic strategy through which to inhibit Aβ deposition in the elderly, thus preventing or delaying the onset of AD.

## Data Availability

The data presented in this study are available upon request from the corresponding author.

## Supplementary Materials

The following supporting information can be downloaded at: https://www.mdpi.com/article/10.3390/nu15194244/s1, Table S1: Macro and micronutrient composition of AIN-93M; Table S2: The fatty acid profile of corn oil.

## References

[1] Zhang X.X., Tian Y., Wang Z.T., Ma Y.H., Tan L., Yu J.T. (2021). The Epidemiology of Alzheimer’s Disease Modifiable Risk Factors and Prevention. J. Prev. Alzheimers Dis..

[2] Ashrafian H., Zadeh E.H., Khan R.H. (2021). Review on Alzheimer’s disease: Inhibition of amyloid beta and tau tangle formation. Int. J. Biol. Macromol..

[3] Wilkins H.M., Swerdlow R.H. (2017). Amyloid precursor protein processing and bioenergetics. Brain Res. Bull..

[4] Scheltens P., De Strooper B., Kivipelto M., Holstege H., Chetelat G., Teunissen C.E., Cummings J., van der Flier W.M. (2021). Alzheimer’s disease. Lancet.

[5] Cheng Y., Tian D.Y., Wang Y.J. (2020). Peripheral clearance of brain-derived Abeta in Alzheimer’s disease: Pathophysiology and therapeutic perspectives. Transl. Neurodegener..

[6] Breijyeh Z., Karaman R. (2020). Comprehensive Review on Alzheimer’s Disease: Causes and Treatment. Molecules.

[7] Scheltens P., Blennow K., Breteler M.M., de Strooper B., Frisoni G.B., Salloway S., Van der Flier W.M. (2016). Alzheimer’s disease. Lancet.

[8] Scarmeas N. (2013). Mediterranean diet improves cognition: The PREDIMED-NAVARRA randomised trial. J. Neurol. Neurosurg. Psychiatry.

[9] Fischer K., Melo van Lent D., Wolfsgruber S., Weinhold L., Kleineidam L., Bickel H., Scherer M., Eisele M., van den Bussche H., Wiese B. (2018). Prospective Associations between Single Foods, Alzheimer’s Dementia and Memory Decline in the Elderly. Nutrients.

[10] Xu Q., Zhang Y., Zhang X., Liu L., Zhou B., Mo R., Li Y., Li H., Li F., Tao Y. (2020). Medium-chain triglycerides improved cognition and lipid metabolomics in mild to moderate Alzheimer’s disease patients with APOE4(-/-): A double-blind, randomized, placebo-controlled crossover trial. Clin. Nutr..

[11] Power R., Nolan J.M., Prado-Cabrero A., Roche W., Coen R., Power T., Mulcahy R. (2022). Omega-3 fatty acid, carotenoid and vitamin E supplementation improves working memory in older adults: A randomised clinical trial. Clin. Nutr..

[12] Watanabe S., Tsujino S. (2022). Applications of Medium-Chain Triglycerides in Foods. Front. Nutr..

[13] Lee Y.Y., Tang T.K., Chan E.S., Phuah E.T., Lai O.M., Tan C.P., Wang Y., Ab Karim N.A., Mat Dian N.H., Tan J.S. (2022). Medium chain triglyceride and medium-and long chain triglyceride: Metabolism, production, health impacts and its applications—A review. Crit. Rev. Food Sci. Nutr..

[14] Hwang C.Y., Choe W., Yoon K.S., Ha J., Kim S.S., Yeo E.J., Kang I. (2022). Molecular Mechanisms for Ketone Body Metabolism, Signaling Functions, and Therapeutic Potential in Cancer. Nutrients.

[15] Achanta L.B., Rae C.D. (2017). beta-Hydroxybutyrate in the Brain: One Molecule, Multiple Mechanisms. Neurochem. Res..

[16] Mett J. (2021). The Impact of Medium Chain and Polyunsaturated omega-3-Fatty Acids on Amyloid-beta Deposition, Oxidative Stress and Metabolic Dysfunction Associated with Alzheimer’s Disease. Antioxidants.

[17] Yin J.X., Maalouf M., Han P., Zhao M., Gao M., Dharshaun T., Ryan C., Whitelegge J., Wu J., Eisenberg D. (2016). Ketones block amyloid entry and improve cognition in an Alzheimer’s model. Neurobiol. Aging..

[18] Lauritzen L., Brambilla P., Mazzocchi A., Harslof L.B., Ciappolino V., Agostoni C. (2016). DHA Effects in Brain Development and Function. Nutrients.

[19] Sinclair A.J. (2019). Docosahexaenoic acid and the brain- what is its role?. Asia Pac. J. Clin. Nutr..

[20] Pan Y., Morris E.R., Scanlon M.J., Marriott P.J., Porter C.J.H., Nicolazzo J.A. (2018). Dietary docosahexaenoic acid supplementation enhances expression of fatty acid-binding protein 5 at the blood-brain barrier and brain docosahexaenoic acid levels. J. Neurochem..

[21] Martinsen A., Saleh R.N.M., Chouinard-Watkins R., Bazinet R., Harden G., Dick J., Tejera N., Pontifex M.G., Vauzour D., Minihane A.M. (2023). The Influence of APOE Genotype, DHA, and Flavanol Intervention on Brain DHA and Lipidomics Profile in Aged Transgenic Mice. Nutrients.

[22] Arellanes I.C., Choe N., Solomon V., He X., Kavin B., Martinez A.E., Kono N., Buennagel D.P., Hazra N., Kim G. (2020). Brain delivery of supplemental docosahexaenoic acid (DHA): A randomized placebo-controlled clinical trial. EBioMedicine.

[23] Kosti R.I., Kasdagli M.I., Kyrozis A., Orsini N., Lagiou P., Taiganidou F., Naska A. (2022). Fish intake, n-3 fatty acid body status, and risk of cognitive decline: A systematic review and a dose-response meta-analysis of observational and experimental studies. Nutr. Rev..

[24] Pifferi F., Dorieux O., Castellano C.A., Croteau E., Masson M., Guillermier M., Van Camp N., Guesnet P., Alessandri J.M., Cunnane S. (2015). Long-chain n-3 PUFAs from fish oil enhance resting state brain glucose utilization and reduce anxiety in an adult nonhuman primate, the grey mouse lemur. J. Lipid Res..

[25] Zhang Y.P., Lou Y., Hu J., Miao R., Ma F. (2018). DHA supplementation improves cognitive function via enhancing Abeta-mediated autophagy in Chinese elderly with mild cognitive impairment: A randomised placebo-controlled trial. J. Neurol. Neurosurg. Psychiatry.

[26] Ardanaz C.G., Ramirez M.J., Solas M. (2022). Brain Metabolic Alterations in Alzheimer’s Disease. Int. J. Mol. Sci..

[27] Ryu J.C., Zimmer E.R., Rosa-Neto P., Yoon S.O. (2019). Consequences of Metabolic Disruption in Alzheimer’s Disease Pathology. Neurotherapeutics.

[28] Cunnane S.C., Trushina E., Morland C., Prigione A., Casadesus G., Andrews Z.B., Beal M.F., Bergersen L.H., Brinton R.D., de la Monte S. (2020). Brain energy rescue: An emerging therapeutic concept for neurodegenerative disorders of ageing. Nat. Rev. Drug. Discov..

[29] Loizzo S., Rimondini R., Travaglione S., Fabbri A., Guidotti M., Ferri A., Campana G., Fiorentini C. (2013). CNF1 increases brain energy level, counteracts neuroinflammatory markers and rescues cognitive deficits in a murine model of Alzheimer’s disease. PLoS ONE.

[30] Puchalska P., Crawford P.A. (2021). Metabolic and Signaling Roles of Ketone Bodies in Health and Disease. Annu. Rev. Nutr..

[31] Cunnane S.C., Courchesne-Loyer A., Vandenberghe C., St-Pierre V., Fortier M., Hennebelle M., Croteau E., Bocti C., Fulop T., Castellano C.A. (2016). Can Ketones Help Rescue Brain Fuel Supply in Later Life? Implications for Cognitive Health during Aging and the Treatment of Alzheimer’s Disease. Front. Mol. Neurosci..

[32] Li K., Wei Q., Liu F.F., Hu F., Xie A.J., Zhu L.Q., Liu D. (2018). Synaptic Dysfunction in Alzheimer’s Disease: Abeta, Tau, and Epigenetic Alterations. Mol. Neurobiol..

[33] Deng Y., Wang Z., Wang R., Zhang X., Zhang S., Wu Y., Staufenbiel M., Cai F., Song W. (2013). Amyloid-beta protein (Abeta) Glu11 is the major beta-secretase site of beta-site amyloid-beta precursor protein-cleaving enzyme 1(BACE1), and shifting the cleavage site to Abeta Asp1 contributes to Alzheimer pathogenesis. Eur. J. Neurosci..

[34] Leong Y.Q., Ng K.Y., Chye S.M., Ling A.P.K., Koh R.Y. (2020). Mechanisms of action of amyloid-beta and its precursor protein in neuronal cell death. Metab. Brain Dis..

[35] Chia P.Z., Gleeson P.A. (2011). Intracellular trafficking of the beta-secretase and processing of amyloid precursor protein. IUBMB Life.

[36] Sawmiller D., Koyama N., Fujiwara M., Segawa T., Maeda M., Mori T. (2023). Targeting apolipoprotein E and N-terminal amyloid beta-protein precursor interaction improves cognition and reduces amyloid pathology in Alzheimer’s mice. J. Biol. Chem..

[37] Mao P., Manczak M., Calkins M.J., Truong Q., Reddy T.P., Reddy A.P., Shirendeb U., Lo H.H., Rabinovitch P.S., Reddy P.H. (2012). Mitochondria-targeted catalase reduces abnormal APP processing, amyloid beta production and BACE1 in a mouse model of Alzheimer’s disease: Implications for neuroprotection and lifespan extension. Hum. Mol. Genet..

[38] Mett J., Lauer A.A., Janitschke D., Griebsch L.V., Theiss E.L., Grimm H.S., Koivisto H., Tanila H., Hartmann T., Grimm M.O.W. (2021). Medium-Chain Length Fatty Acids Enhance Abeta Degradation by Affecting Insulin-Degrading Enzyme. Cells.

[39] Camberos-Luna L., Geronimo-Olvera C., Montiel T., Rincon-Heredia R., Massieu L. (2016). The Ketone Body, beta-Hydroxybutyrate Stimulates the Autophagic Flux and Prevents Neuronal Death Induced by Glucose Deprivation in Cortical Cultured Neurons. Neurochem. Res..

[40] Green K.N., Martinez-Coria H., Khashwji H., Hall E.B., Yurko-Mauro K.A., Ellis L., LaFerla F.M. (2007). Dietary docosahexaenoic acid and docosapentaenoic acid ameliorate amyloid-beta and tau pathology via a mechanism involving presenilin 1 levels. J. Neurosci..

[41] Borsini A., Stangl D., Jeffries A.R., Pariante C.M., Thuret S. (2020). The role of omega-3 fatty acids in preventing glucocorticoid-induced reduction in human hippocampal neurogenesis and increase in apoptosis. Transl. Psychiatry.

[42] Lazarev V.F., Tsolaki M., Mikhaylova E.R., Benken K.A., Shevtsov M.A., Nikotina A.D., Lechpammer M., Mitkevich V.A., Makarov A.A., Moskalev A.A. (2021). Extracellular GAPDH Promotes Alzheimer Disease Progression by Enhancing Amyloid-beta Aggregation and Cytotoxicity. Aging. Dis..

[43] Minoshima S., Cross D., Thientunyakit T., Foster N.L., Drzezga A. (2022). (18)F-FDG PET Imaging in Neurodegenerative Dementing Disorders: Insights into Subtype Classification, Emerging Disease Categories, and Mixed Dementia with Copathologies. J. Nucl. Med..

[44] Smailagic N., Vacante M., Hyde C., Martin S., Ukoumunne O., Sachpekidis C. (2015). (1)(8)F-FDG PET for the early diagnosis of Alzheimer’s disease dementia and other dementias in people with mild cognitive impairment (MCI). Cochrane Database Syst. Rev..

[45] Dave A., Hansen N., Downey R., Johnson C. (2020). FDG-PET Imaging of Dementia and Neurodegenerative Disease. Semin. Ultrasound CT MR..

[46] Minoshima S., Mosci K., Cross D., Thientunyakit T. (2021). Brain [F-18]FDG PET for Clinical Dementia Workup: Differential Diagnosis of Alzheimer’s Disease and Other Types of Dementing Disorders. Semin. Nucl. Med..

[47] Mosconi L., Murray J., Davies M., Williams S., Pirraglia E., Spector N., Tsui W.H., Li Y., Butler T., Osorio R.S. (2014). Nutrient intake and brain biomarkers of Alzheimer’s disease in at-risk cognitively normal individuals: A cross-sectional neuroimaging pilot study. BMJ Open.

[48] Roy M., Nugent S., Tremblay-Mercier J., Tremblay S., Courchesne-Loyer A., Beaudoin J.F., Tremblay L., Descoteaux M., Lecomte R., Cunnane S.C. (2012). The ketogenic diet increases brain glucose and ketone uptake in aged rats: A dual tracer PET and volumetric MRI study. Brain Res..

