# Supplementary material for: Supplementation of Medium-Chain Triglycerides Combined with Docosahexaenoic Acid Inhibits Amyloid Beta Protein Deposition by Improving Brain Glucose Metabolism in APP/PS1 Mice
Source: Nutrients. 2023 Oct 1;15(19):4244. doi: 10.3390/nu15194244 (PMC10574179; doi:10.3390/nu15194244)
Supplement: Supplementary file 1 [file nutrients-15-04244-s001.zip › nutrients-2589700-supplementary.pdf]

Supplementary Materials

Table S1. Macro and micronutrient composition of AIN-93M

Items	AIN-93M
Energy	3601 kcal/kg
Water content	6.8%
Fat	4%
Carbohydrates	72.7%
Protein	12.5%
Fatty acids profile	
SFA	16.87%
C14:0	0.06%
C16:0	10.65%
C17:0	0.05%
C18:0	4.86%
C20:0	0.20%
C21:0	0.61%
C22:0	0.34%
C24:0	0.09%
MUFA	23.71%
C16:1	0.07%
C17:1	0.03%

C18:1 (n-9)	23.44%
C20:1 (n-9)	0.17%
N-6 PUFA	53.67%
C18:2 (n-6)	53.07%
C18:3 (n-6)	0.60%
N-3 PUFA	5.76%
C18:3 (n-3)	5.75%
Mineral content	
Calcium	5000 ppm
Phosphorus	3000 ppm
Potassium	3600 ppm
Sodium	1033 ppm
Magnesium	511 ppm
Iron	45 ppm
Zinc	35 ppm
Fierce	10 ppm
Copper	6 ppm
Iodine	0.2 ppm
Chromium	1 ppm
Inorganic sulfur	300 ppm
Chlorine	1613 ppm
Vitamin content	

Vitamin A	4 IU/g
Vitamin D	1 IU/g
Vitamin E	0.075 IU/g
Vitamin K	0.86 ppm
Thiamine, B1	5 ppm
Riboflavin	6 ppm
Nicotinic acid	30 ppm
Pantothenic acid	15 ppm
Vitamin B6	6 ppm
Choline	1000 ppm
Folic acid	2 ppm
Biotin	0.2 ppm
Vitamin B12	25 ppb

Note: Abbreviations: ppm, part per million; IU, International Unit; ppb, part per billion.

Table S2. The fatty acid profile of corn oil

**Fatty acid**	**Corn oil**
SFA	19.48%
C12:0	0.26%
C14:0	1.22%
C16:0	13.42%
C18:0	3.34%
C20:0	0.43%
MUFA	32.26%
C16:1 (n-7)	0.26%
C18:1 (n-9)	31.00%
C18:1 (n-7)	0.65%
C20:1 (n-9)	0.26%
N-6 PUFA	47.34%
C18:2 (n-6)	47.22%
N-3 PUFA	1.24%
C18:3 (n-3)	1.24%
